# Editorial on Child Abuse and Neglect II

**DOI:** 10.3390/children12060694

**Published:** 2025-05-28

**Authors:** Eva Möhler

**Affiliations:** Department of Child and Adolescent Psychiatry, Saarland University Medical Center, 66421 Homburg, Germany; eva.moehler@uks.eu

This second Special Issue in *Children* on child abuse and neglect continues the intense scientific debates on the prevention, assessment, and treatment of different forms of maltreatment against children. The Adverse Childhood experiences study [1] was one of the first to demonstrate the detrimental long-term effects of childhood adversity on biological and psychosocial health.

Previous studies have repeatedly focused on topics such as the effects of child abuse and neglect on mental, physical, and social health [1]; epigenetic aging [2]; intergenerational transmission [3]; and brain structure alteration [4]. The fact that individuals subjected to childhood maltreatment can have their lifespan shortened by up to 20 years as a result of these factors should be a sufficient indicator of the necessity for further research and development projects centered on the prevention of childhood abuse and its consequences.

This Special Issue contributes to ongoing scientific developments in several ways: The brief intervention named ‘Play Nicely’ is a low-threshold tool focusing on parents’ beliefs about physical punishment. In one study, the sustained effect of this single-session online intervention on the reduction of physical punishment was remarkable in the intervention group, leading to the parental belief that ‘Punishment is the best alternative to control children’s behavior’. An increase in positive parenting could also be observed. This is a very important addition to prevention approaches reported previously [5].

A specific benefit of this article is the demonstration that low-threshold interventions, such as one single online coaching session, can be effective. Since barriers to treatment, specifically in socioeconomically disadvantaged populations, have been described to prevent effective treatment strategies, easy-to-implement- approaches such as ‘Play Nicely’ seem to provide promising novel strategies for the prevention of childhood abuse.

The parental mindset, as an important target of investigation, is also the focus of a study in this Special Issue describing parental stress as an important mediator of physical punishment in families, along with parental beliefs in the efficacy of physical punishment [6]. The identification of targets for prevention, such as parental stress, is an essential aspect of this article as both parental stress and parental beliefs seem to be modifiable, according to the previously mentioned interventions [5].

Parental education on appropriate disciplinary strategies was also called for by a Taiwanese study group reporting on criminal justice cases involving child abuse with mild to severe physical consequences [7]. In this study, lower educated young males who were cohabitating partners of biological parents were most frequently identified as perpetrators, pointing to a specific population target for prevention.

A case report proposed a new diagnostic entity—the so-called ‘compressed baby head’—thereby broadening the scope of diagnosis in abusive head trauma. Abusive trauma to an infant’s head has frequently been reported as the so-called ‘shaken baby syndrome’, indicating that shaking is the only form of violence directed toward an infant’s head. One article presented in this Special Issue proposes the idea of adding a new diagnostic entity resulting from compression trauma of the head rather than the shaking of an infant, and the authors present multiple medical findings to support their proposal [8].

A new perspective of fundamental importance is presented by the authors of [9], who analyzed child abuse in sports, presenting results at the microsystem level and considering factors such as forced training, dehydration, violence from coaches and peers, and emotional abuse with and without verbal attacks. The authors identify excessive parental trust in coaches and their inadequate supervision, as well as a lack of response to abuse by sports clubs, as contributing and aggravating factors. They also report critical levels of normalizing abuse, specifically affecting female athletes.

On this note, and in a more general perspective, child protection concepts [10], seem mandatory, not only in sports clubs, but also in child care facilities and schools. Further studies on the development and implementation of child-protective measures such as participation and other evaluated strategies, such as those reviewed by [7], will be highly important for future research.
